# Complete Genome Sequences of Six Staphylococcus pseudintermedius Strains from Dogs with Superficial Pyoderma in Georgia, USA

**DOI:** 10.1128/MRA.00378-21

**Published:** 2021-06-24

**Authors:** Maurice Byukusenge, Frane Banovic, Lingling Li, Suresh V. Kuchipudi, Bhushan M. Jayarao, Cynthia K. Watson, Hemant K. Naikare

**Affiliations:** a School of Veterinary Medicine, University of Rwanda, Nyagatare, Rwanda; b Department of Small Animal Medicine and Surgery, College of Veterinary Medicine, University of Georgia, Athens, Georgia, USA; c Animal Diagnostic Laboratory, Pennsylvania State University, University Park, Pennsylvania, USA; d Center for Infectious Disease Dynamics, Pennsylvania State University, University Park, Pennsylvania, USA; e Tifton Veterinary Diagnostic and Investigational Laboratory, College of Veterinary Medicine, University of Georgia, Tifton, Georgia, USA; University of Delaware

## Abstract

Staphylococcus pseudintermedius is a pathogen of veterinary importance, as it is the major causative agent of superficial pyoderma in dogs. We present the complete genome sequences of six strains of S. pseudintermedius derived from dogs affected with epidermal collarettes and superficial bacterial folliculitis, which are two variants of superficial pyoderma.

## ANNOUNCEMENT

Superficial pyoderma is a common diagnosis in the dog, with a prevalence of up to 10% in all dogs presented in private practices (1, 2). Staphylococcus pseudintermedius is the principal pathogen for canine superficial pyoderma (3). Pathogenic S. pseudintermedius strains are underrepresented in genomic databases such as GenBank compared to human pathogens. Here, we present the complete genome sequences of six strains of S. pseudintermedius, which were isolated from dogs in Georgia, USA, suffering from two clinical variants of superficial pyoderma, epidermal collarettes (strains 9261-1A, 11304-1A, 11304-2A, 11304-3A, and 11306-1A) and superficial bacterial folliculitis (strain 11306-4A). Two plasmids were assembled from the sequencing data from a single strain (11304-3A). These complete genome sequences will improve researchers’ capacity for using genomic data for an in-depth understanding of the mechanisms underlying the pathogenesis of S. pseudintermedius-mediated superficial pyoderma clinical variants.

Swab samples were collected from dogs affected with superficial pyoderma at the Veterinary Medical Center of the College of Veterinary Medicine, University of Georgia. The Institutional Animal Care and Use Committee (IACUC) of the University of Georgia (CR-459) approved the study protocol. The sample swabs were inoculated onto blood agar (tryptic soy agar with 5% sheep blood; Thermo Fisher Scientific) and incubated for 24 h at 37 ± 2°C in an aerobic incubator. Preliminary identification of isolated colonies was made using conventional methods (i.e., positive catalase test, positive coagulase test) and the Gram-positive organism bacterial auto-identification system (Sensititre; Thermo Fisher Scientific) according to the manufacturer’s procedure. The species identification of S. pseudintermedius was performed using the multiplex PCR method for species identification of coagulase-positive staphylococci using the bacterial DNA from single bacterial colonies (4). Pure isolated colonies of S. pseudintermedius strains isolated from canine superficial pyoderma were transported to the Penn State Animal Diagnostic Laboratory for sequencing. The single isolated colonies were subcultured in brain heart infusion (BHI) broth (BD) and incubated overnight at 37°C. DNA extraction from each colony was done using the GenFind V2 DNA extraction kit (Beckman Coulter) following the manufacturer’s instructions.

Two platforms were used for sequencing; Illumina was utilized to generate short paired-end reads, whereas long reads for closing the gaps in the genomic sequences were generated with the MinION device from Oxford Nanopore Technologies (ONT). The Illumina Nextera DNA Flex library prep kit was used to generate the libraries for sequencing with Illumina MiniSeq. The one-dimensional (1D) native barcoding genomic DNA protocol (EXP-NBD104 and SQK-LSK109; ONT) was used to generate the libraries for sequencing with the MinION device. The quality of the short reads (150 bp) generated with Illumina MiniSeq was assessed using FastQC v0.11.9 (5). Base-calling and demultiplexing of reads from the MinION data were performed using MinKNOW v20.10 and EPI2ME v2020.2.10, respectively; both software platforms are downloadable from the ONT community website. Filtlong v0.2.0 (6) was used for quality control by removing short reads and trimming off the regions of the lowest quality from each read. A *de novo* hybrid assembly technique with Unicycler v0.4.8 (7) was utilized with default options to generate complete circular chromosomes for all six isolates and two plasmids for one isolate. The sequence overlap identification and trimming and the genome rotation are among Unicycler’s default options. The circular replicons are rotated to start with *dnaA* and *repA* genes for chromosomes and plasmids, respectively. The genome sequences were submitted to GenBank and annotated using the NCBI Prokaryotic Genome Annotation Pipeline (PGAP) (8). The metric sequencing data and genome assembly are provided in Table 1.

**TABLE 1:** Metrics for sequence data and accession numbers of six genomes of S. pseudintermedius from Georgia

Strain	Replicon type	Genome size (bp)	GC content (%)	Total no. of genes	GenBank accession no.	BioSample accession no.	Data for Illumina sequencing			Data for Oxford Nanopore sequencing		
							Total reads (bp)	Avg read length (bp)	Avg coverage (x)	Total reads (bp)	*N*_50_ (bp)	Avg coverage (x)
SP_11304-1A	Chromosome	2,558,081	37.7	2,445	CP065925.1	SRS7863626	2,046,244	147.2	118	37,415	5,423	70
SP_11304-2A	Chromosome	2,605,960	37.7	2,475	CP065924.1	SRS7863627	2,233,820	147.9	127	55,623	5,211	100
SP_11304-3A	Chromosome	2,610,514	37.6	2,496	CP065921.1	SRS7863628	2,199,510	147.6	123	47,417	6,456	99
	Plasmid 1	15,734	28.7	17	CP065922.1							
	Plasmid 2	3,348	31.2	3	CP065923.1							
SP_11306-1A	Chromosome	2,605,463	37.6	2,468	CP065920.1	SRS7863629	2,341,202	147.8	133	58,587	4,824	100
SP_11306-4A	Chromosome	2,539,776	37.8	2,394	CP065919.1	SRS7863625	2,052,768	149.9	120	28,339	9,466	102
SP_9261-1A	Chromosome	2,671,923	37.5	2,530	CP065926.1	SRS7863624	1,467,696	147.6	81	61,446	4,477	97

### Data availability.

The data were deposited in the NCBI’s databases under the BioProject accession no. PRJNA683859. The complete genomes and the raw reads were deposited in the GenBank and SRA databases, respectively. The accession numbers are provided in Table 1.
